# Immunostimulatory Effect of Heat-Killed Probiotics on RAW264.7 Macrophages

**DOI:** 10.4014/jmb.2201.01015

**Published:** 2022-03-20

**Authors:** Hye-Ji Noh, Jung Min Park, Yoo Jin Kwon, Kyunghwan Kim, Sung Yurb Park, Insu Kim, Jong Hyun Lim, Byoung Kook Kim, Byung-Yong Kim

**Affiliations:** 1 Probiotics Research Laboratory, Chong Kun Dang Bio Research Institute (CKDBIO), Gyeonggi 15064, Republic of Korea; 2 R&D Center, Chong Kun Dang Healthcare (CKDHC), Seoul 07249, Republic of Korea

**Keywords:** Immunostimulatory effect, macrophages, heat-killed probiotics

## Abstract

Probiotics modulate the gut microbiota, which in turn regulate immune responses to maintain balanced immune homeostasis in the host. However, it is unclear how probiotic bacteria regulate immune responses. In this study we investigated the immunomodulatory effects of heat-killed probiotics, including *Lactiplantibacillus plantarum* KC3 (LP3), *Lactiplantibacillus plantarum* CKDB008 (LP8), and *Limosilactobacillus fermentum* SRK414 (LF4), via phagocytosis, nitric oxide (NO), and pro-inflammatory cytokine production in macrophages. We thus found that heat-killed LP8 could promote the clearance of foreign pathogens by enhancing the phagocytosis of macrophages. Treatment with heat-killed LP8 induced the production of NO and pro-inflammatory cytokines, including TNF-α, IL-6, and IL-1β. In addition, heat-killed LP8 suppressed the production of NO and cytokines in LPS-induced RAW264.7 cells, suggesting that heat-killed LP8 exerts immunomodulatory effects depending on the host condition. In sum, these results indicate that heat-killed LP8 possesses the potential for immune modulation while providing a molecular basis for the development of functional probiotics prepared from inactivated bacterial cells.

## Introduction

The host defense mechanism of innate immunity is the first line of defense in mediating protection against infection [1]. In this context, macrophages are important cellular components of the innate immune system, as they secrete immunoregulatory mediators that enable an adaptive immune response to protect against foreign pathogens in the host [2, 3]. Meanwhile, toll-like receptors (TLRs) constitute a key signaling pathway in modulating macrophage function; in conjunction with microbial components, including lipopolysaccharides (LPS), they trigger the activation of intracellular signaling cascades such as mitogen-activated protein kinases (MAPKs) and the nuclear factor *kappa*-light-chain-enhancer of activated B cells (NF-κB), which leads to macrophage activation [4]. In turn, activated macrophages enhance phagocytosis, promote the production of cytotoxic molecules, including nitric oxide (NO), and secrete immunoregulatory cytokines (including tumor necrosis factor-α (TNF-α), interleukin (IL)-1β, IL-6, and others) to initiate the destruction of pathogens [5]. As such, macrophages are key target cells for immunomodulatory mechanisms. As components of the normal gut intestinal flora, probiotics support a balanced immune system [6]. Indeed, many studies have reported on their efficacy in promoting health [7-9]. They are formally defined as “live microorganisms which when administered in adequate amounts confer a health benefit on the host” (FAO/WHO 2002). In the immunomodulatory context, probiotics regulate gut microbiota, which interact with other host immune systems [10, 11]. Despite the many health benefits, there are still some concerns about the safety of using probiotics, as side effects may emerge due to the diverse properties of living microbial cells (FAO/WHO 2002) [12-14]. With the aim of avoiding these risks, the research trend is gradually shifting from probiotics to inactivated bacterial cells (parabiotics) and/or their metabolites (postbiotics) [15-17].

While the scientific concept that non-living microorganisms can improve host health is not new, a variety of terminologies have been employed to date, including “heat-killed probiotics,” “postbiotics,” “parabiotics,” and “ghost probiotics.” Moreover, there is a large body of research on the immunomodulatory activities of bioactive compounds produced by probiotics, but inactivated probiotics are now emerging as promising immune-boosting supplements [16, 18-20]. As one of the various inactivated forms, heat-killed probiotics contain nonviable bacterial cells and/or metabolites produced by live probiotics. Both the components of probiotic cells (*e.g.*, exopolysaccharides (EPS), peptidoglycans, and lipoteichoic acid) and metabolites (*e.g.*, short-chain fatty acids and amino acids) also improve immune systems and metabolic disorders. For example, EPS derived from *Lacticaseibacillus casei* WXD030 can modulate immune responses by enhancing proliferation and phagocytic activity while inducing the production of NO and cytokines in macrophages [21]. Kwon *et al*. reported that EPS isolated from *Lactiplantibacillus plantarum* L-14 inhibited pro-inflammatory mediators, including those of the NF-κB and MAPK pathways, by suppressing TLR4 and MyD88 signaling in macrophages [22]. Likewise, research has shown that peptidoglycans from gram-positive probiotic bacteria, especially *Lactobacillus acidophilus* and *Lacticaseibacillus rhamnosus*, can regulate LPS-induced inflammation and inhibit the release of inflammatory cytokines in macrophages [23]. Although there is evidence on the anti-inflammatory activities of some bioactive components derived from probiotics, there has been a lack of discussion on their immunostimulatory activities and molecular mechanisms.

As such, in this study we evaluated the potential immunoregulatory properties of three new heat-killed probiotics, including *Lactiplantibacillus plantarum* KC3 (LP3), *Lactiplantibacillus plantarum* CKDB008 (LP8), and *Limosilactobacillus fermentum* SRK414 (LF4), in RAW264.7 macrophages, which constitutes a critical strategy for protecting and enhancing immune function.

## Materials and Methods

### Materials

Lipopolysaccharides (LPS *Escherichia coli* O111:B4 and O55:B5) were purchased from Sigma-Aldrich (USA). TNF-α, IL-6, and IL-1β enzyme-linked immunosorbent assay (ELISA) kits were purchased from R&D Systems (USA) and BioLegend (USA). The NO detection kits (cat. ADI-917-010) were purchased from Enzo Life Sciences (USA).

### Preparing the Heat-Killed Probiotics

The two *Lactiplantibacillus* (formerly *Lactobacillus*) *plantarum* strains LP3 and LP8 were isolated from Korean homemade baechoo-kimchi, a traditional Korean fermented food, while the *Limosilactobacillus* (formerly *Lactobacillus*) *fermentum* strain LF4 was isolated from newborn baby feces. The species of the isolated strains were identified via 16S rRNA gene sequencing (SolGent Co., Ltd. Korea) using the EzBioCloud database (https://www.ezbiocloud.net/). Following this, the selected strains were cultivated and maintained in a developed culture broth at 37°C for 16-24 h. To evaluate their immunoregulatory effects, the cultivated strains were heat-killed at 121°C for 20 min. The heat-killed probiotics LP3, LP8, and LF4 were then harvested via centrifugation. Finally, the whole-cell lysates were freeze-dried and used for further experimentation.

### Cell Culture

The murine macrophage cell line RAW264.7 was obtained from the Korean Cell Line Bank (KCLB, Korea) and the American Type Culture Collection (ATCC TIB-71; ATCC, USA). The macrophages were maintained in Dulbecco’s modified Eagle medium (DMEM; Gibco, USA), as supplemented with 10% (v/v) fetal bovine serum (FBS; Gibco) and 1% penicillin/streptomycin (Gibco) at 37°C in a humidified 5% CO_2_ incubator. The cells were subcultured and plated at 70-80% confluency.

### Phagocytic Activity

The CytoSelect 96-Well Phagocytosis Assay (zymosan substrate) Kit (Cell Biolabs. Inc., USA) was used to evaluate phagocytic activity in the microphages, as accomplished via the zymosan substrate included in the kit. All experiments were performed in accordance with the manufacturer’s protocol.

### Measuring NO Production

RAW264.7 macrophages (4 × 10^4^ cells/well) were seeded in 96-well plates for 24 h and then starved in DMEM supplemented with 1% FBS overnight before treatment. After starvation, the cells were pre-incubated with 1 μg/ml LPS for 4 h, followed by treatments with different concentrations of the heat-killed probiotics LP3, LP8, and LF4 for 24 h. The supernatant was then collected, with NO production determined using an NO detection kit in accordance with the manufacturer’s protocol.

### Western Blot Analysis

After cells were harvested, cell lysates were extracted in 1X RIPA buffer (50 mM Tris (pH8.0), 1% NP40, 0.25%sodium deoxycholic acid, 150 mM NaCl, 1 mM EDTA, and complete mini-protease cocktail). Cell lysates (40 μg) were separated using 10% SDS-PAGE before being transferred to Immune Blot TM NC membranes (Bio-Rad, USA) and blocked with 5% skim milk (BD Bioscience, USA). Primary antibodies targeted inducible nitric oxide synthase (iNOS) and β-actin. Horseradish peroxidase-conjugated secondary antibodies against mouse were obtained from Santa Cruz Biotechnology. Protein expression was measured using a Chemstudio imager (Analytic Jena, USA) and quantified using VisionWorks software. The protein bands were visualized using an enhanced chemiluminescence solution (PerkinElmer, USA).

### Measuring Cytokines

RAW264.7 cells were cultured at a density of 5 × 10^5^ cells/well in 24-well plates in triplicate with various concentrations (1×10^7^, 5×10^7^, and 1×10^8^ CFU/ml) of the heat-killed probiotics LP3, LP8, and LF4, with or without LPS. After 24h of incubation, released cytokine levels (TNF-α, IL-6, and IL-1β) in the culture supernatant were measured using commercially available R&D Systems and BioLegend products in accordance with the manufacturers’ instructions.

### Statistical Analysis

The results were presented as means ± SDs. Statistical significance was analyzed via Student’s *t*-test using IBM SPSS (version 22.0; IBM Corp., USA) to test for mean differences among the samples. Intergroup differences were considered statistically significant at *p* < 0.05.

## Results

### Identification of Bacterial Strains

As a result of 16S rRNA gene sequence analysis and comparison with the type strain of each species, LP3 was identified as *Lactiplantibacillus plantarum* with a similarity of 99%; LP8 was identified as *L. plantarum* with a similarity of 99%, and LF4 was identified as *Limosilactobacillus fermentum* with a similarity of 99% (Table 1).

### Effects of Heat-Killed Probiotics on Phagocytic Activities in RAW264.7 Cells

**Fig. 1:**
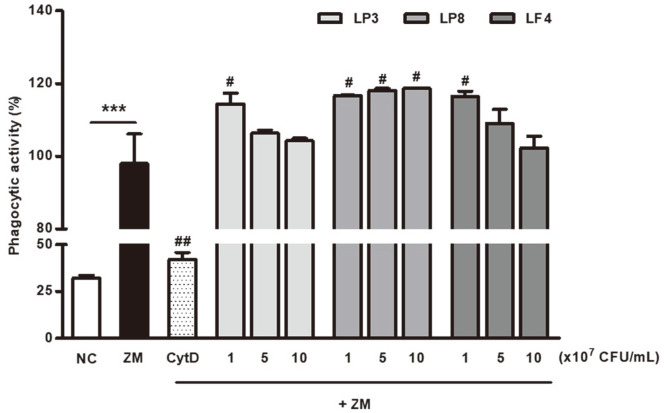
Effects of heat-killed probiotics on the phagocytosis of RAW264.7 cells. Data were expressed as the percent phagocytosis rates of macrophages in the presence of heat-killed probiotics, compared with treated ZM (zymosan) cells, taken as 100%. All data are presented as means ± SDs. *p <0.05, **p <0.01, and ***p <0.001 indicate significant differences from the NC, while #*p* <0.05, ##*p* <0.01, and ###*p* <0.001 indicate significant differences from the ZM control. NC (negative control), cell only; ZM, zymosan; CytD, 1 μM cytochalasin D+zymosan; LP3, *Lactiplantibacillus plantarum* KC3+zymosan; LP8, *Lactiplantibacillus plantarum* CKDB008+zymosan; LF4, *Limosilactobacillus fermentum* SRK414+zymosan.

Phagocytic activity, including that induced by macrophages, is the first step in providing host defense against pathogens, and is an important part of the innate immune system [24]. As phagocytosis is a biological function of macrophages in the elimination of specific pathogens, tumor cells, and damaged cells, we investigated the effects of heat-killed LP3, LP8, and LF4 on phagocytic activity, which was monitored by measuring the amount of internalized zymosan particles in macrophages. As shown in Fig. 1, the highest activity was observed for LP8, followed by LF4 and LP3, respectively (*p* <0.05). The phagocytic activity of the LP8-treated group was significantly and dose-dependently increased by up to 119% at 1 × 10^8^CFU/ml when compared to the zymosan control (*p* < 0.05). LP3 and LF4 significantly enhanced the phagocytic activity of macrophages when compared to the zymosan control, but not in a concentration-dependent manner. This suggests that heat-killed LP8 particularly enhances the phagocytic activity of macrophages, thereby boosting immune function.

### Effects of Heat-Killed Probiotics on NO Production and iNOS Expression in LPS-Induced RAW264.7 Macrophage Cells

**Fig. 2:**
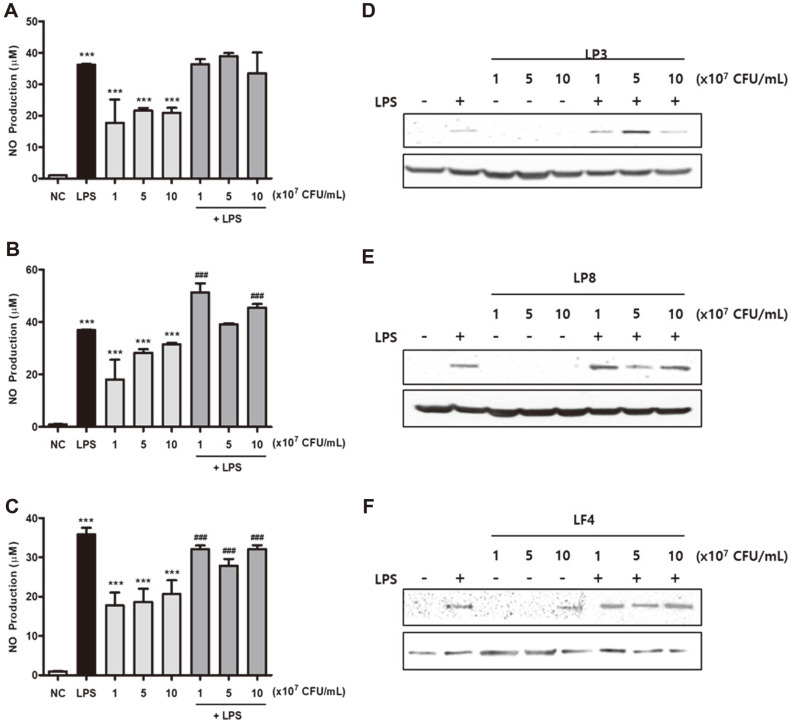
Effects of heat-killed probiotics on NO production and iNOS protein expression in LPS-induced RAW264.7 cells. Cells were pretreated with 1 μg/ml in DMEM containing 1% LPS conditions for 4 h, then exposed to the heat-killed probiotics LP3, LP8, and LF4 in a dose-dependent manner for 24 h. NO production was determined from culture media (A, B, and C), while iNOS expression was analyzed by western blotting (D, E, and F). *p <0.05, **p <0.01, and ***p <0.001 indicate significant differences from the NC, while #*p* <0.05, ##*p* <0.01, and ###*p* <0.001 indicate significant differences from the LPS control. NC (negative control), cell only; LPS, lipopolysaccharide; LP3, *Lactiplantibacillus plantarum* KC3; LP8, *Lactiplantibacillus plantarum* CKDB008; LF4, *Limosilactobacillus fermentum* SRK414.

We observed that LP3, LP8, and LF4 had anti-inflammatory abilities that were at least partially due to decreased NO and iNOS levels (data not shown). We also investigated the effects of LP3, LP8, and LF4 on the production of cytokines related to inflammation, with treatment concentrations selected based on the MTT assay results (Figs. S1A-S1C). Briefly, the cells were treated with LPS for 4 h, followed by LP3, LP8, and LF4 treatments for 24 h. Culture media were collected and used to determine the production of NO; cells were lysed with RIPA buffer to collect protein samples. In sum, the LP3, LP8, and LF4 treatments significantly increased the production of NO when compared to the control (Figs. 2A-2C). It is well known that NO production initiates through iNOS induction via cytokines; in this study, heat-killed probiotics reduced LPS-induced iNOS protein expression in a concentration-dependent manner. In other words, LP3, LP8, and LF4 reduced the LPS-induced elevation of iNOS protein expression. Of particular note, iNOS protein levels were not affected by either the LP3 or LP8 treatments, as compared to the control (Figs. 2D-2F).

### Effects of Heat-Killed Probiotics on Cytokine Secretion

**Fig. 3:**
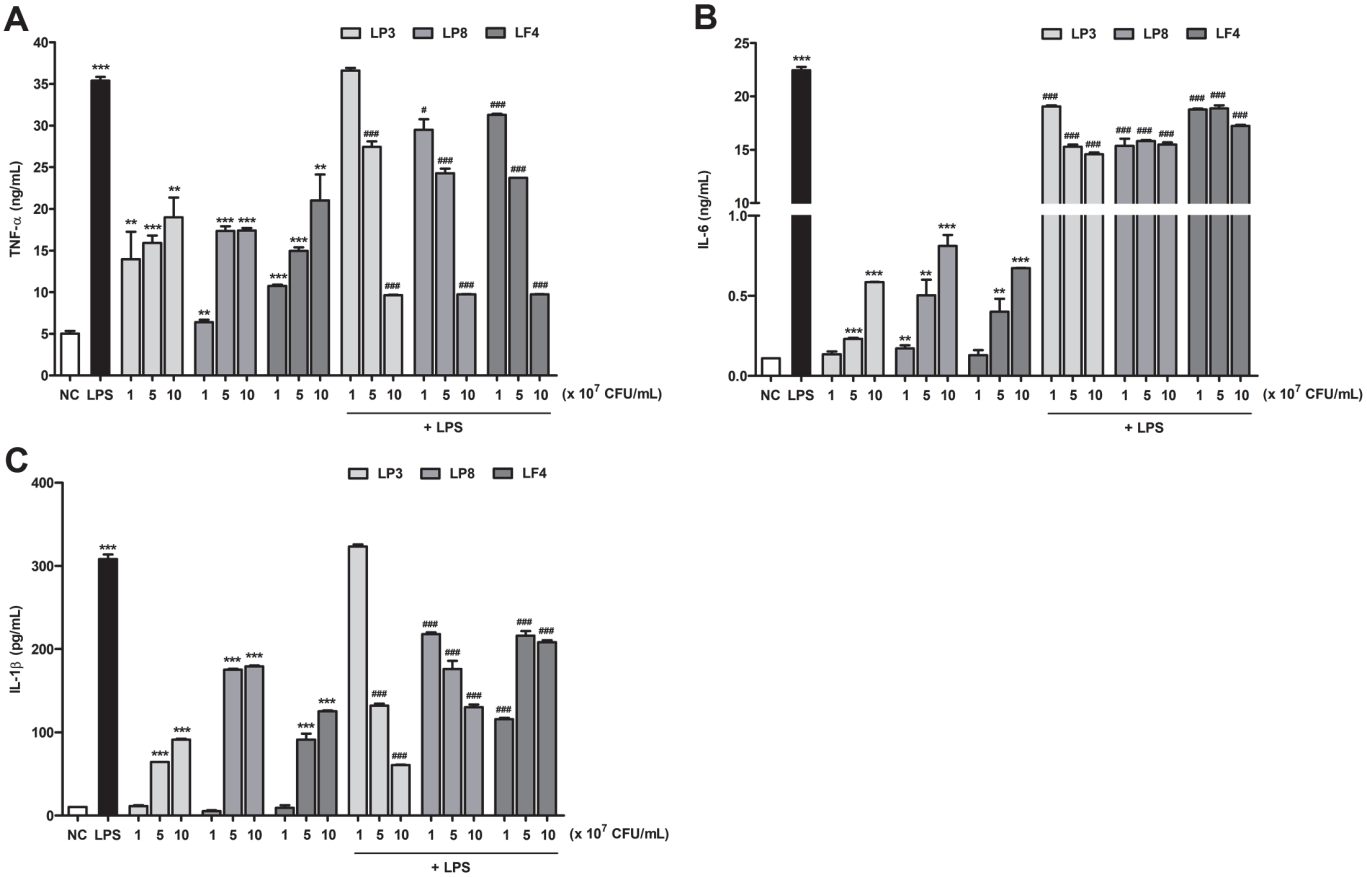
Effects of heat-killed probiotics on cytokine production by RAW 264.7 cells. Cells were treated with different concentrations of the heat-killed probiotics LP3, LP8, and LF4 or stimulated with LPS for 24 h. TNF-α (**A**), IL-6 (**B**), and IL-1β (**C**) production was measured via ELISA. All data are presented as means ± SDs. *p <0.05, **p <0.01, and ***p <0.001 indicate significant differences from the NC, while #*p* <0.05, ##*p* <0.01, and ###*p* <0.001 indicate significant differences from the LPS control. NC (negative control), cell only; LPS, lipopolysaccharide (100 ng/ml); LP3, *Lactiplantibacillus plantarum* KC3; LP8, *Lactiplantibacillus plantarum* CKDB008; LF4, *Limosilactobacillus fermentum* SRK414.

Cytokines play important roles in regulating inflammatory and immune responses. Those such as TNF-α, IL-6, and IL-1β are also potent immunomodulators of activated macrophages [25]. To further investigate whether heat-killed, probiotics-activated RAW264.7 cells influenced the production of cytokines in the presence or absence of LPS stimulation, we collected culture supernatants at 24 h and then measured the amounts of TNF-α, IL-6, and IL-1β via ELISA. As shown in Fig. 3, the heat-killed, probiotics-treated cells showed increased TNF-α, IL-1β, and IL-6 secretion levels when compared to the NC group in a dose-dependent manner. Among the three tested heat-killed probiotics, LP8 most prominently stimulated cytokine production via the activated macrophages. Moreover, none of the heat-killed probiotics were toxic to cell viability within the tested concentration range (data not shown). These results suggest that heat-killed LP3, LP8, and LF4 exert immune-enhancing effects via cytokine upregulation in activated macrophages. Meanwhile, the LPS-treated cells showed significantly higher TNF-α, IL-1β, and IL-6 production when compared to the NC group (*p* < 0.05). However, the heat-killed LP3, LP8, and LF4 significantly inhibited the production of TNF-α, IL-1β, and IL-6 in LPS-stimulated cells with increasing concentrations, thus suggesting a shift toward anti-inflammatory status (*p* < 0.05, Fig. 3). In sum, LP8 showed more effective cytokine regulation in both the presence and absence of LPS stimulation when compared to that imposed by other heat-killed probiotics.

## Discussion

Heat-killed probiotics contain soluble products and metabolites that are either secreted by live bacteria or released after bacterial lysis, thus providing physiological benefits to the host [26]. Recently, in vitro and in vivo studies have reported that some heat-killed probiotics exhibit physiological benefits. For example, the administration of heat-killed *Lacticaseibacillus casei* DKGF7 for a period of four weeks helped improve symptoms in an animal IBS model [18]. Further, the daily oral intake of heat-killed *Lactiplantibacillus plantarum* L-137 for a period of 12 weeks significantly decreased total cholesterol, low-density lipoprotein cholesterol, and leukocyte counts in overweight individuals [19]. However, there is not much evidence on macrophage-related immune modulation via heat-killed probiotics, nor is there sufficient information on their mechanisms of action. To address these gaps in the literature, we investigated the immunomodulatory effects of heat-killed probiotics, namely LP3, LP8, and LF4 in vitro and attempted to clarify the underlying mechanisms that contribute to the health benefits of heat-killed probiotics.

The innate immune response is the first line of defense against invading pathogens. Previous research suggests that macrophages play essential roles in helping the innate immune system recognize foreign pathogens [2]. Macrophages can detect and clear invading pathogens and apoptotic cells through phagocytosis, which plays essential roles in innate immunity and inflammation control [27]. In turn, macrophage activation is a major event in innate immunity, particularly for initiating and propagating defensive reactions against pathogens. In this context, phagocytosis is the first macrophagic response against invading microorganisms, and therefore heightens the innate immune response. In this study we found that heat-killed LP3, LP8, and LF4 each induced the phagocytic activity of macrophages, with LP8 inducing the highest levels among all three types. These results suggest that heat-killed LP8 can stimulate the activation of immune cells by triggering the phagocytic activity of macrophages. Similarly, other studies have shown that heat-killed *Lacticaseibacillus casei* IMAU60214 promotes the phagocytic activity of macrophages via TLR2 activation in vitro [28] while *L. brevis* KCCM 12203P enhances phagocytosis in *E. coli* particles in macrophages [29]. This is the first report to confirm that heat-killed LP8 activates the phagocytic activity of macrophages, which is the first step in inducing innate immunity.

Immune responses are initiated and perpetuated by molecules derived from pathogen-associated molecular patterns (PAMPs) of microorganisms or from the damage or death of host cells’ damage-associated molecular patterns (DAMPs). PAMPs and DAMPs are recognized by membrane-bound or vesicular (endosomal) pattern-recognition receptors (PRRs), including the Toll-like receptors (TLRs) [30]. PAMP/DAMP recognition by PRRs triggers the activation of innate immune cells such as macrophages, which then induce the production of NO and cytokines, including TNF-α, IL-6, and IL-1β [25]. Moreover, NO is produced by iNOS in activated macrophages during phagocytosis and is an important bactericidal factor against pathogens in host defense mechanisms [31]. Further, TNF-α, IL-6, and IL-1β are produced by activated macrophages, lymphocytes, endothelial cells, and fibroblasts, and may induce increased vascular permeability while recruiting inflammatory cells that mediate the transition from innate to adaptive immunity [25]. The inflammatory response is beneficial for the host when the aforementioned cytokines are produced in appropriate amounts, but becomes toxic when produced in a deregulated manner [25].

Pro-inflammatory mediators (including NO, TNF-α, IL-6, and IL-1β, which are produced in appropriate amounts by activated macrophages) also play key roles in controlling both the inflammatory state and immune responses in the host. In this study we found that heat-killed LP3, LP8, and LF4 significantly enhanced the production of NO and cytokines (including TNF-α, IL-6, and IL-1β) in LPS-treated macrophages in a dose-dependent manner. By contrast, heat-killed LP3, LP8, and LF4 significantly decreased NO and cytokine production (including TNF-α, IL-6, and IL-1β) in LPS-treated macrophages. These results suggest that heat-killed LP3, LP8, and LF4 may help increase immunity or suppress inflammatory responses, depending on the host condition. Of all three types, LP8 showed the most prominent effects. Lee *et al*. revealed that treatment with dead nano-sized *L. plantarum* in RAW264.7 cells stimulated NO production and iNOS expression more than live *L. plantarum*, and that the stimulatory properties were probably largely derived from its cell wall [32]. Similarly, LP8 stimulated higher NO and cytokine production in RAW264.7 cells compared to that of live *L. plantarum* in our study (data not shown). The immunostimulatory effects of LP8 indicate that LP8 could help activation of macrophages by heat-killed probiotic cell wall, cell-free supernatants, and components derived from probiotics.

In conclusion, we found that LP8 exerted an immunomodulatory effect on RAW264.7 macrophage cells. It appears that these effects resulted from macrophage activation, as evidenced by activated phagocytosis and the regulation of NO and pro-inflammatory cytokines. In this regard, future in-depth research should aim to delineate the contributions of signaling pathways to the immune-regulatory efficacy of LP8 within the current model. Regardless of these limitations, the current findings suggest that LP8 may enhance immunity when used as a food ingredient or otherwise taken as a dietary supplement.

## Supplemental Materials

Supplementary data for this paper are available on-line only at http://jmb.or.kr.

## Figures and Tables

**Table 1:** Species identification of the bacterial strains.

Strain	Species identification	Identity (%)	Reference strain no.	Isolation source
LP3	*Lactiplantibacillus plantarum*	99	BS404	Fermented kimchi
LP8	*Lactiplantibacillus plantarum*	99	JCM 1149	Fermented kimchi
LF4	*Limosilactobacillus fermentum*	99	NBRC 15885	Newborn infant
