# Supplementary material for: Immunostimulatory Effect of Heat-Killed Probiotics on RAW264.7 Macrophages
Source: J Microbiol Biotechnol. 2022 Mar 20;32(5):638–44. doi: 10.4014/jmb.2201.01015 (PMC9628881; doi:10.4014/jmb.2201.01015)
Supplement: Supplementary file 1 [file jmb-32-5-638-supple.pdf]

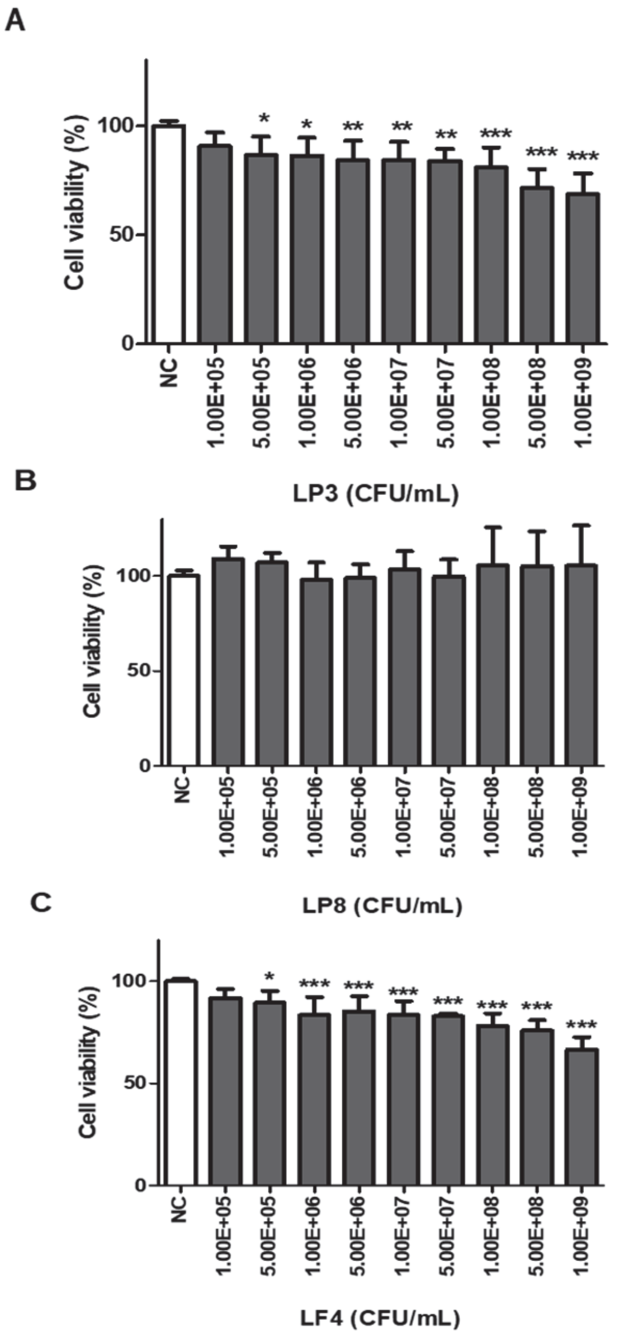

Fig. S1. Effects of heat-killed probiotics on the viability of RAW 264.7 cells

RAW 264.7 macrophages were seeded in 96-well plates for 24 hours, followed by 18 hours of starvation. Cells were treated with LP3 (A), LP8 (B), or LF4 (C) in a dose-dependent manner for 24 hours. Cell viability was measured using the MTT assay and quantified as a percentage (%) of that of the control. Data are presented as means ± standard deviations (SD). * p <0.05 versus the control.
